# *Toxoplasma* Co-opts Host Cells It Does Not Invade

**DOI:** 10.1371/journal.ppat.1002825

**Published:** 2012-07-26

**Authors:** Anita A. Koshy, Hans K. Dietrich, David A. Christian, Jason H. Melehani, Anjali J. Shastri, Christopher A. Hunter, John C. Boothroyd

**Affiliations:** 1 Department of Medicine (Infectious Disease), Stanford University School of Medicine, Stanford, California, United States of America; 2 Department of Neurology, Stanford University School of Medicine, Stanford, California, United States of America; 3 Department of Pathobiology, School of Veterinary Medicine, University of Pennsylvania, Philadelphia, Pennsylvania, United States of America; 4 Department of Microbiology & Immunology, Stanford University School of Medicine, Stanford, California, United States of America; UMDNJ-New Jersey Medical School, United States of America

## Abstract

Like many intracellular microbes, the protozoan parasite *Toxoplasma gondii* injects effector proteins into cells it invades. One group of these effector proteins is injected from specialized organelles called the rhoptries, which have previously been described to discharge their contents only during successful invasion of a host cell. In this report, using several reporter systems, we show that *in vitro* the parasite injects rhoptry proteins into cells it does not productively invade and that the rhoptry effector proteins can manipulate the uninfected cell in a similar manner to infected cells. In addition, as one of the reporter systems uses a rhoptry:Cre recombinase fusion protein, we show that in Cre-reporter mice infected with an encysting *Toxoplasma*-Cre strain, uninfected-injected cells, which could be derived from aborted invasion or cell-intrinsic killing after invasion, are actually more common than infected-injected cells, especially in the mouse brain, where *Toxoplasma* encysts and persists. This phenomenon has important implications for how *Toxoplasma* globally affects its host and opens a new avenue for how other intracellular microbes may similarly manipulate the host environment at large.

## Author Summary

*Toxoplasma gondii* is an intracellular parasite that infects warm blooded animals, including humans. In these hosts, *Toxoplasma* establishes a chronic infection in the brain, which the parasite accomplishes in part by injecting effector proteins, which manipulate many cellular processes, into cells it invades. Two recent reports suggested that *Toxoplasma* may also inject effector proteins into cells it does not invade. To look for these “uninfected-injected” cells, we utilized three different reporter systems that are tied to injection of effector proteins and not to invasion. With these systems, we determined that *Toxoplasma* injects proteins into cells it does not invade and enough protein is injected to manipulate the uninfected cells in a manner consistent with what occurs in infected cells. Furthermore, by using one of the reporter systems in mice, we verified that these uninfected-injected cells can include systemic immune cells and neurons in the brain. Remarkably, in the brain, the uninfected-injected cells out-number the infected cells by many fold. Together, these results strongly suggest that *Toxoplasma* manipulates far more cells than previously realized and, given their abundance, these uninfected-injected cells may play a central role in how *Toxoplasma* engages the host's immune response.

## Introduction

Obligate intracellular organisms, from viruses to eukaryotic pathogens, modify the microenvironment of the infected host cell to avoid clearance by host-cell-intrinsic mechanisms (e.g., autophagy, phago-lysosomal fusion) as well as to block the immune system from recognizing the host cell as infected. One commonly deployed method utilized by cellular pathogens is to secrete effector proteins which modify the cell or the cellular compartment in which the pathogen resides– such as LLO from *Listeria monocytogenes*, AvrA from *Salmonella*, or ROP16 from *Toxoplasma* [1]–[3]. Unlike extracellular gram-negative bacteria which can potentially target effector proteins to cells distant from their location [4], obligate intracellular pathogens have previously only been known to secrete their effector proteins into the cell in which they reside [5]. The presumption has been that invasion or uptake is required in order to initiate injection or secretion of these effector proteins but two recent reports on *Toxoplasma gondii*, an obligate intracellular parasite related to *Plasmodium*, have challenged this notion [6], [7].

*Toxoplasma* is a protozoan parasite that has a broad host range and is known to be capable of invading almost any nucleated cell [8]. The tachyzoite invasion process is associated with gross manipulation of host cell processes, including immune response genes, carbohydrate metabolism, and apoptosis [3], [9], [10]. Although all the details are not yet known, recent studies have shown that a significant portion of this manipulation is initiated less than one minute into the invasion process [11], during which time *Toxoplasma* injects effector proteins into the host cell. Many of these effector proteins originate from specialized, apically-localized organelles called rhoptries [12]. Recent reports have provided evidence that some injected rhoptry proteins affect host transcription factors and block cell-intrinsic defense mechanisms [3], [13].

The molecular details of how rhoptry proteins are injected are unknown; *Toxoplasma* has no homologs of any of the well-studied bacterial secretions systems nor has a candidate secretion apparatus been identified. And although, until recently, all the evidence suggested that rhoptry discharge only occurred in cells in which the parasite invaded, it should be noted that immediately after invasion, a few of the dozen or so rhoptries still appear “full”, suggesting that not all are discharged during the invasion process and that the parasite has enough loaded rhoptries for multiple invasion attempts [14].

In a productive invasion event, which includes establishment of a parasitophorous vacuole (PV) and subsequent replication within that PV, *Toxoplasma* tachyzoites must first strongly attach to the cell. Interestingly, *in vitro*, it has been described that only about a quarter of these strong attachments result in invasion [15]. It has been unclear whether this low rate of invasion simply reflects an inefficient process or whether this might even be a selected trait that serves some other purpose. Potentially, during these abortive events, *Toxoplasma* might be probing the cell to determine if it is optimal for invasion. Alternatively, it could be that the parasite has evolved to deliberately inject a bolus of effector proteins into cells it does not intend to invade. Consistent with this latter hypothesis, by infecting fibroblasts that only express eGFP after Cre-mediated recombination with parasites engineered to express Cre fused to the rhoptry protein “toxofilin”, we recently reported that eGFP expression could be observed in both infected cells as well as in a nearly equal number of cells that do not contain parasites [6], [16]. Similar observations have recently been made using *in vitro Toxoplasma* infections of primary macrophages where SOCS3 up-regulation was seen in both infected and uninfected cells [7].

While the data from both studies are consistent with injection without invasion, they could also be explained by cell division after invasion with one daughter cell receiving the single PV and the other emerging “uninfected”. Alternatively, the SOCS3 upregulation could result from local paracrine effects of factors secreted by the infected cell or even cell-to-cell communication of inflammatory signals, which was recently described in HeLa cells infected with *Salmonella* [17]. To discriminate between these possibilities and to specifically assess whether *Toxoplasma* can inject rhoptry effector proteins into cells it does not invade, we sought to determine the origins of these uninfected-manipulated cells (cells injected with *Toxoplasma* protein but not containing a parasite) as well as to determine whether or not such cells could be found *in vivo*. Using a combination of reporter systems, we show that while some of the uninfected-manipulated cells do appear to result from division of the host cell after invasion, a significant fraction do not. *In vivo*, the effect is particularly striking in the brains of infected mice.

## Results

### Infected cultures of Cre-reporter cells show evidence of rhoptry injection in cells that do not contain parasites

**Figure 1:**
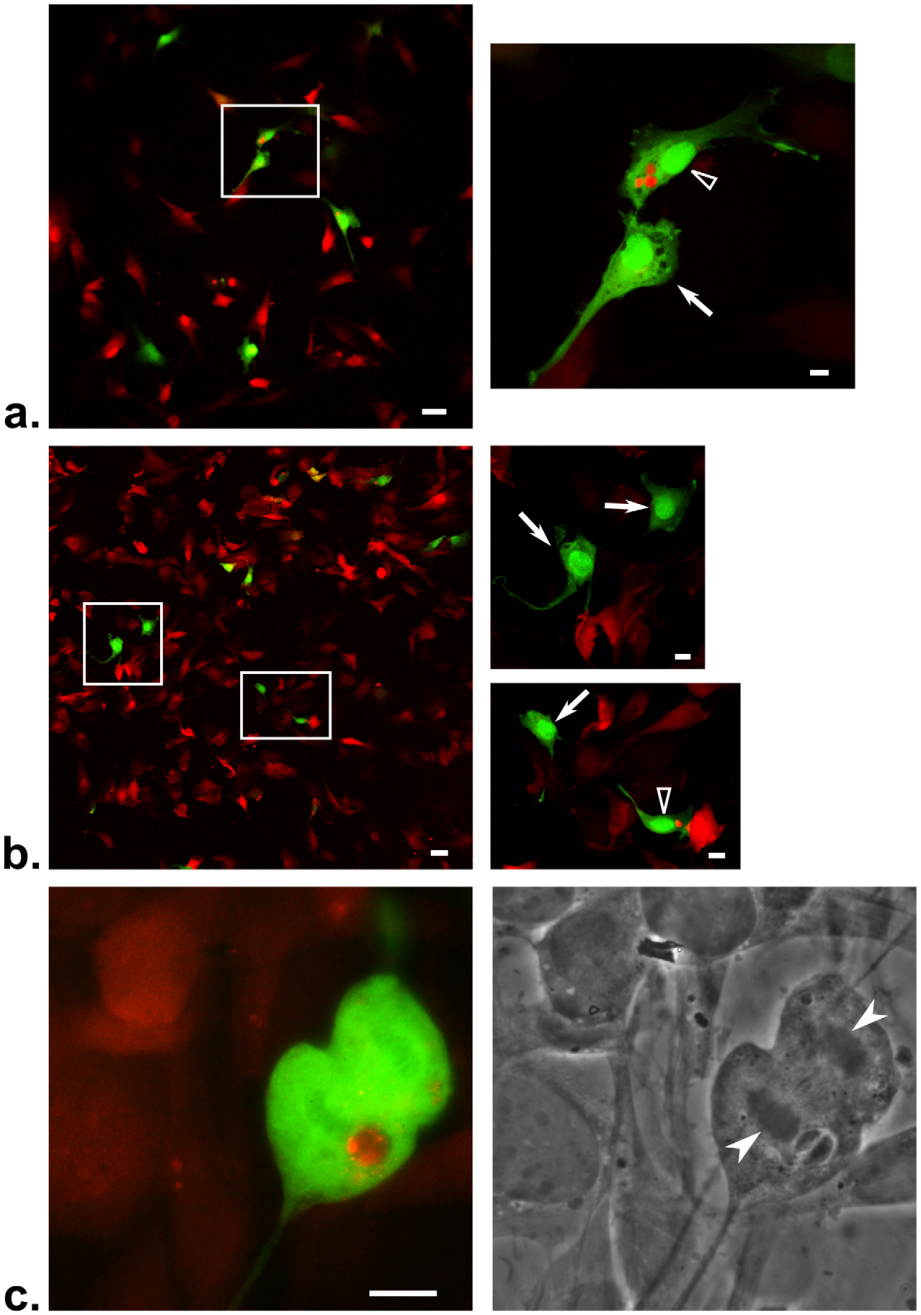
Cultures of Cre-reporter cells infected with Toxoplasma-Cre strains show 2 distinct populations of uninfected-injected cells (U-I cells). Cre-reporter fibroblasts were incubated for 24 hours with *Toxoplasma*-Cre parasites at an MOI of 0.5, fixed and then examined by fluorescence microscopy. (a) Merged (red and green) image of a Pru-mCherry-Cre strain showing an infected (empty arrowhead) and adjacent, uninfected (arrow) eGFP^+^ host cell. Right panel is an enlargement of the boxed areas in the left panel. Red derives both from the DsRed of cells without Cre-mediated recombination and the mCherry expressed by the parasites. Green represents eGFP expression in a cell after Cre-mediated recombination. Scale bar = 50 µm for left panel and 10 µm for right panel(s). (b) As for (a) except uninfected eGFP^+^ cells that are not adjacent to infected cells are shown. (c) As for (a) except a host cell in the midst of division is shown. In this case, a non-mCherry expressing RH-Cre strain was used as the infecting strain and anti-SAG1 antibodies were used to visualize the parasites. The left panel shows a color merge where red represents both anti-SAG1 staining of the parasites and DsRed of the host cells and green derives from eGFP fluorescence; the right panel shows the corresponding phase image. Note the condensed nuclei (white filled arrowheads) of the dividing host cell. Scale bar = 10 µm.

**Figure 2:**
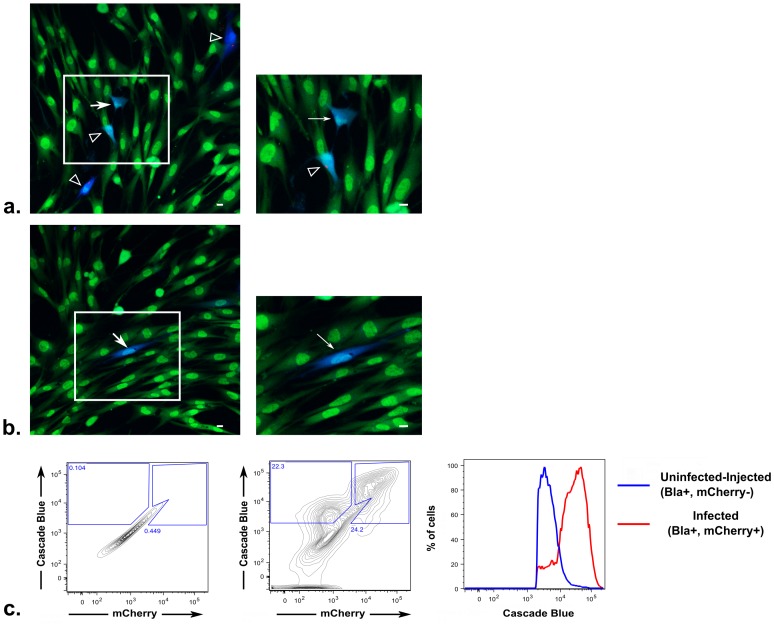
Human foreskin fibroblast (HFFs) cultures show U-I cells after incubation with *Toxoplasma*-β-lactamase strain. HFFs were plated at near-confluence and incubated with a RH-mCherry-β-lactamase strain at an MOI of 0.5 for 2 hours prior to loading for 30 minutes with the detection substrate (CCF2-AM). Live images were obtained using a confocal microscope. Green and blue fluorescence represent the uncleaved and cleaved CCF2-AM substrate, respectively; red represents mCherry expression by the parasites. (a) and (b) are representative color-merged images from the same experiment. White arrows indicate cells with substrate cleavage (blue) but no detectable parasite. Empty arrowheads indicate infected cells with substrate cleavage. Right panels are enlarged views of boxed regions in the left panels. Scale bars = 10 µm. (c) Flow cytometry analysis of fully confluent cultures incubated with a RH-mCherry-β-lactamase strain at an MOI of 0.5 for 2 hours prior to substrate loading. Panels from left to right represent contour plots of unstained, uninfected HFF cultures used to draw gates for blue^+^, mCherry^−^ and blue^+^, mCherry^+^ cells; gating applied to the contour plot of HFFs incubated with the RH-mCherry-β-lactamase strain and loaded with CCF2-AM; and the histogram of the prior panel comparing the mean fluorescence intensity of blue of the U-I cells (upper-left gate) compared to infected cells (upper-right gate).

In our previous report, where we first observed uninfected-manipulated cells, we employed a commonly used laboratory strain of *Toxoplasma* (RH). This highly virulent strain is unable to encyst and cause a latent infection in mice thereby severely complicating *in vivo* studies. To facilitate *in vivo* studies, therefore, we engineered a less virulent, encysting *Toxoplasma* strain (Pru) to express mCherry as well as the previously described rhoptry-targeted Cre fusion protein, toxofilin:Cre [6]. We then used the resulting Pru-mCherry-Cre strain to infect the previously described Cre-reporter fibroblasts at a multiplicity of infection (MOI) of 0.5. Twenty four hours post-infection (hpi), the cultures were examined by fluorescence microscopy to detect cells that had undergone Cre-mediated recombination and therefore expressed eGFP. In addition to the expected eGFP-positive, infected host cells (Figure 1 a,b), we observed eGFP-positive, uninfected cells at a rate of about 40–50% of the frequency of the infected green cells. These uninfected green cells fell into two categories: (1) those in contact with or in very close proximity to an infected host cell (Figure 1a) or (2) those that were not in direct contact with and were distant from infected host cells (Figure 1b). While the latter population is further from infected host cells, an infected cell was always seen within a radius of 5–10 cells from the green uninfected cell (data not shown). *A priori*, these uninfected green cells observed during *Toxoplasma*-Cre infections could arise by several possible mechanisms: (a) the reporter cells exhibit a background leakiness (i.e., expression of eGFP occurs without undergoing Cre-mediated recombination), (b) the reporter cells take up either plasmid DNA or the Cre fusion protein from the extracellular environment; (c) after invasion, the host cells clear the parasites by cell-intrinsic mechanisms, (d) a host cell undergoes cell division after invasion and only one daughter acquires the PV, and/or (e) invasion is initiated, including injection of the rhoptry proteins, but the process is aborted.

We previously showed that background leakiness can be excluded as an explanation for the uninfected green cells because no eGFP-expressing cells are found in Cre-reporter cultures in the absence of infection or upon infection with live parasites that do not inject Cre [6]. Similarly, we were able to exclude uptake of Cre fusion protein/DNA from the extracellular environment as an explanation because no eGFP-expressing cells were observed after incubation with heat-killed *Toxoplasma*-Cre parasites, freeze-thaw lysates of such parasites, or “conditioned” media from cultures heavily infected with these parasites [6]. We also examined reporter cells into which the toxofilin-Cre plasmid had been transfected (without parasites) and again saw no eGFP expression indicating that even if some amount of the parasite DNA is taken up, the toxofilin:Cre fusion protein will not be expressed [6].

Destruction of parasites after invasion is also unlikely to be the explanation for the uninfected eGFP-positive cells as we did not stimulate the cultures with cytokines and IFN-γ is a known requirement for non-immune cells to initiate cell-intrinsic defense mechanisms against *Toxoplasma* [18], [19]. Also, in none of these experiments did we find mCherry debris or partially destroyed parasites in the Cre-reporter cells, which is consistent with these cells lacking the ability to destroy invaded parasites without cytokine stimulation. This left cell division after invasion and/or injection without invasion as possible explanations for the uninfected green cells. We know that host cell division can occur after Cre-mediated recombination secondary to parasite invasion as we see occasional green cells in the process of dividing (Figure 1c). This, then, likely explains many of the uninfected green cells that lie in contact with infected cells. Cell division, however, does not explain the uninfected green cells that are separated from infected host cells by many uninfected cells as the reporter cells being used are non-motile fibroblasts and so, daughter cells should lie next to each other, not several cells apart. Taken in total, the results described above support but do not prove the hypothesis that a portion of the uninfected green cells are likely produced from *Toxoplasma* injecting its rhoptry proteins without proceeding to invade the cell.

### Primary fibroblast cultures show evidence of early rhoptry protein injection into cells without parasites

The Cre/*loxP* assay is extremely sensitive and binary; even one functional Cre tetramer has the potential to catalyze excision at the *loxP* sites and, once removed, expression of the reporter is not further influenced by more Cre being introduced. Hence, this assay provides little indication as to the amount of Cre introduced. To address this shortcoming and to confirm that the phenomenon of uninfected-injected cells is not dependent upon the immortalized nature of the Cre-reporter cells, we repeated our analysis using primary host cells (human foreskin fibroblasts) and a *Toxoplasma* strain expressing a toxofilin:β-lactamase fusion protein [20]. The β-lactamase assay uses a substrate that emits light at different wavelengths depending on whether it has been cleaved by β-lactamase, and the ratio of cleaved to uncleaved substrate determines the relative intensity of signal detected at each wavelength [21]. Furthermore, as β-lactamase will immediately cleave the substrate upon contact (as opposed to the Cre-based assay which requires Cre-mediated recombination, transcription of the recombined DNA and translation of the resulting transcripts), the β-lactamase assay allows for the assessment of rhoptry protein injection much sooner after cells have been incubated with parasites. Hence, the β-lactamase-based assay greatly reduces the possibility of reporter-positive, uninfected host cells being due to host cell division or parasite destruction by the host cell following injection of the rhoptry fusion protein. In addition, the β-lactamase assay has a greater dynamic range than the Cre-based assay with the potential ability to detect varying amounts of injected enzyme.

Using β-lactamase-injecting parasites, we infected near-confluent human foreskin fibroblast (HFF) cultures at an MOI of 0.5 for two hours and then added the detection substrate. As anticipated from the results described above and as seen in Figure 2, we could detect the presence of the toxofilin:β-lactamase fusion protein in many cells that did not contain a parasite (uninfected blue cells, arrows). Also consistent with our previous results, we observed two populations of uninfected blue cells: one population that was in contact with infected cells (Figure 2a) and one population that was more distant (Figure 2b). Given the short time period between the incubation period and live cell imaging and the lack of mCherry debris in the uninfected blue cells, it is unlikely that many, if any, of these latter cells arose from parasite clearance by cell-intrinsic mechanisms. For similar reasons and because the host cells were HFFs, which are primary cells that have strong contact inhibition, it is likely that very few of the uninfected blue cells seen by this method were derived from division of infected cells, although this does appear to be the case for some; e.g., the uninfected cell in Figure 2a appears to be connected directly to the infected cell next to it suggesting a recent division has occurred. On the other hand, the blue cell highlighted in Figure 2b, which is completely surrounded by cells that show no evidence of substrate cleavage, is very unlikely to have arisen from the division of an infected cell. Interestingly, many of these uninfected and infected blue cells show a range of blueness. By eye, the uninfected cells do not show a trend of being markedly less blue than the infected cells.

To better quantify the color of the uninfected versus infected blue cells, confluent HFF cultures were treated in the same way as described above except that instead of being imaged by confocal microscopy, the cells were trypsinized and analyzed by flow cytometry. As seen in Figure 2c, the uninfected blue cells show a substantial but somewhat lower mean fluorescence intensity compared to the infected blue cells. These data suggest that compared to the infected blue cells, the uninfected blue cells receive less rhoptry protein, the rhoptry fusion protein is more rapidly degraded in such cells and/or the uninfected cells more efficiently export the cleaved substrate. Regardless, these results show that in primary cells and using a less sensitive assay for rhoptry injection, we can recapitulate the uninfected-injected cell phenomenon we found using the Cre/*loxP* system. In addition, the data provide strong evidence that these uninfected-injected cells receive a substantial amount of rhoptry proteins, although possibly less than the amount introduced into productively invaded cells. Having established by two systems that a portion of these cells most likely arises from aborted invasion, from here forward these cells will be referred to as uninfected-injected (U-I) cells.

### HFF cultures infected with an unmodified, non-encysting *Toxoplasma* strain show that rhoptry injection into uninfected cells involves physiologically relevant amounts

**Figure 3:**
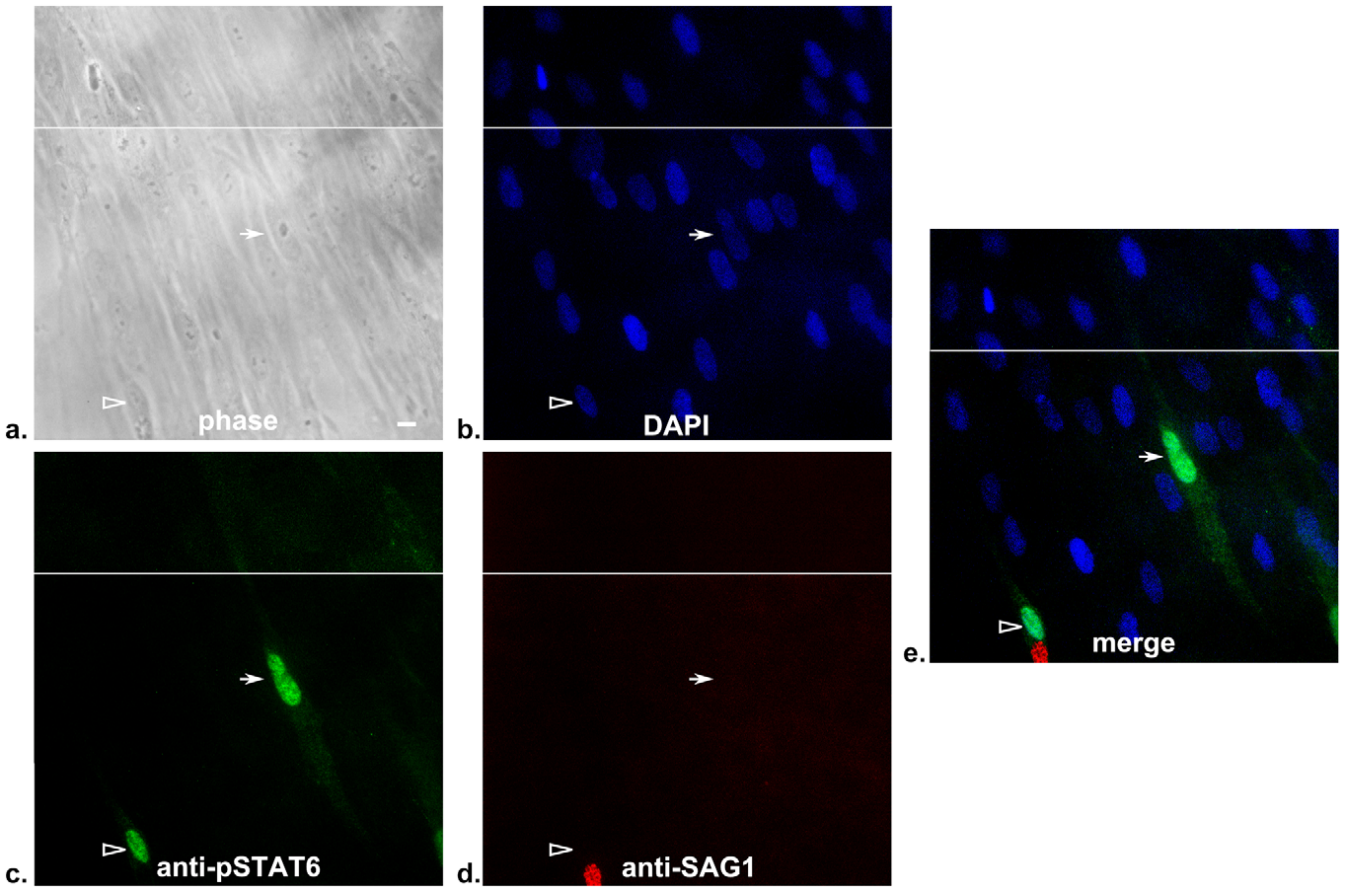
Primary fibroblast cultures show U-I cells with phosphorylation and nuclear translocation of STAT6. Confluent fibroblast cultures were infected at an MOI of 0.02 with wild-type RH strain parasites that express only native rhoptry proteins. After 18 hours, the cultures were fixed, stained, and then examined by fluorescence microscopy. Panels: (a) phase, (b) UV filter (DAPI), (c) green filter (anti-pSTAT6 antibody), (d) red filter (anti-SAG1 antibody), (e) merged image of UV, green, and red filters. Each panel shows two abutting fields to ensure the entirety of the central cell with anti-pSTAT6 staining could be visualized. The white horizontal line indicates where the two fields abut. The empty arrowhead and arrow point to an infected and uninfected cell with activated pSTAT6, respectively. Scale bar = 10 µm.

The previous experiments confirmed that U-I cells could be observed using 2 different reporter methods for detection of rhoptry injection; however, neither experiment enabled us to assess the functional consequence of such a phenomenon. In addition, both assays utilized ectopic expression of the same rhoptry protein (toxofilin) as the base for the fusion proteins. To address if other rhoptry proteins are being injected and, if so, whether they are introduced in physiologically relevant amounts, we took advantage of the fact that injection of the tyrosine kinase rhoptry protein, ROP16, into HFFs invaded by *Toxoplasma* causes rapid phosphorylation and nuclear translocation of STAT6. This phenotype is well documented, important for the host's immune response and is specifically dependent upon the injection of ROP16 into the host cell [3], [11]. Thus, we infected confluent HFF cultures at a low MOI with a wild-type *Toxoplasma* strain that expresses only its native rhoptry proteins. Eighteen hours after infection, we assessed these cultures by immunofluorescence assay (IFA) for phosphorylation and nuclear translocation of STAT6. As seen in Figure 3, we again observed that a significant fraction of cells (∼6%) that show STAT6 phosphorylation and nuclear translocation contain no parasites (arrow). Given the distance between this particular U-I cell and the nearest infected cell (empty arrowhead), it is highly improbable that the U-I cell arose secondary to cell division and then migrated several cells over to its current position. Mock-infected HFF cultures never showed phosphorylation and nuclear translocation of STAT6 (data not shown).

The percentage of U-I cells showing the pSTAT6 phenotype is significantly lower than the percentage of U-I cells found using the Cre-reporter system or the β-lactamase assay; this is not surprising and is likely due to several factors. First, the detection of pSTAT6 is likely to be much less sensitive than either the Cre or β-lactamase reporter assay. Consistent with this decreased sensitivity is the fact that pSTAT6 signal scales with the number of invasion events (e.g., the presence of two invaded parasites is associated with a stronger pSTAT6 signal compared with singly infected cells) [3] while neither of the other reporter assays appeared to scale in this manner [6], [20]. Additionally, as the IFA was done 18 hours after incubation with parasites, it is possible that the pSTAT6 signal may be more robustly maintained in completed invasion events compared to aborted ones (e.g., through the on-going interaction with other parasite effectors released after invasion). Nevertheless, these results with pSTAT6 activation confirm that, *in vitro*, *Toxoplasma* secretes multiple rhoptry proteins into cells it does not invade and that in at least some of these cells the amount of protein injected is sufficient to induce important changes in host cell physiology consistent with what is observed during productive invasion.

### Uninfected, injected cells occur frequently *in vivo* and in immune cells show phosphorylation and nuclear translocation of STAT6

**Figure 4:**
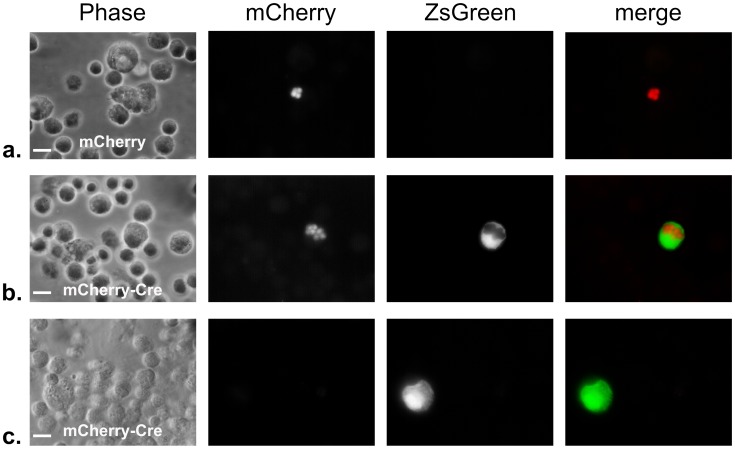
Peritoneal exudate cells from Cre-reporter mice show U-I cells in mice infected with *Toxoplasma*-mCherry-Cre strain. Cre-reporter mice that express ZsGreen only after Cre-mediated recombination were inoculated i.p. with 5000 tachyzoites of either the control Pru-mcherry parasites (a) or the Pru-mcherry-Cre parasites (b,c). 4 days after inoculation, the peritoneal exudate cells (PECs) were removed, fixed, and examined by fluorescence microscopy. Panels from left to right show phase, red filter (parasite mCherry), green filter (host ZsGreen), and merge of the red and green filter images, respectively. Note the infected ZsGreen^+^ cell in (b) and the uninfected ZsGreen^+^ cell in (c). Scale bars = 10 µm.

All of the above studies were done *in vitro* and it could be that the U-I phenomenon is an artifact of suboptimal conditions in tissue culture. Therefore, to verify that U-I cells are also generated during *in vivo* infections, we returned to the Cre/*loxP* assay and utilized Cre-reporter mice that express ZsGreen only after Cre-mediated recombination [22]. We infected these mice with the encysting Pru-mCherry-Cre strain or a control strain, which expresses a non-functional toxofilin:Cre fusion protein as well as mCherry (Pru-mCherry). We then examined the peritoneal exudate cells (PECs) at 4 days post infection (dpi) by fluorescence microscopy. Figure 4 shows that in mice infected with the control strain, no ZsGreen-expressing inflammatory cells are seen (panel a), while in mice infected with the Cre-expressing strain, both infected (panel b) and uninfected (panel c) ZsGreen^+^ inflammatory cells are seen. To exclude the possibility these U-I PECs are secondary to an unknown ability of activated immune cells to take up Cre protein from the extracellular environment (e.g. liberated from killed parasites), we infected a Cre reporter mouse with the control Pru-mCherry strain and 2 dpi injected the mouse intraperitoneally with recombinant Cre protein. The PECs were removed 3 days later (5 dpi) and examined by fluorescence microscopy; no ZsGreen^+^ cells were observed (data not shown.)

**Figure 5:**
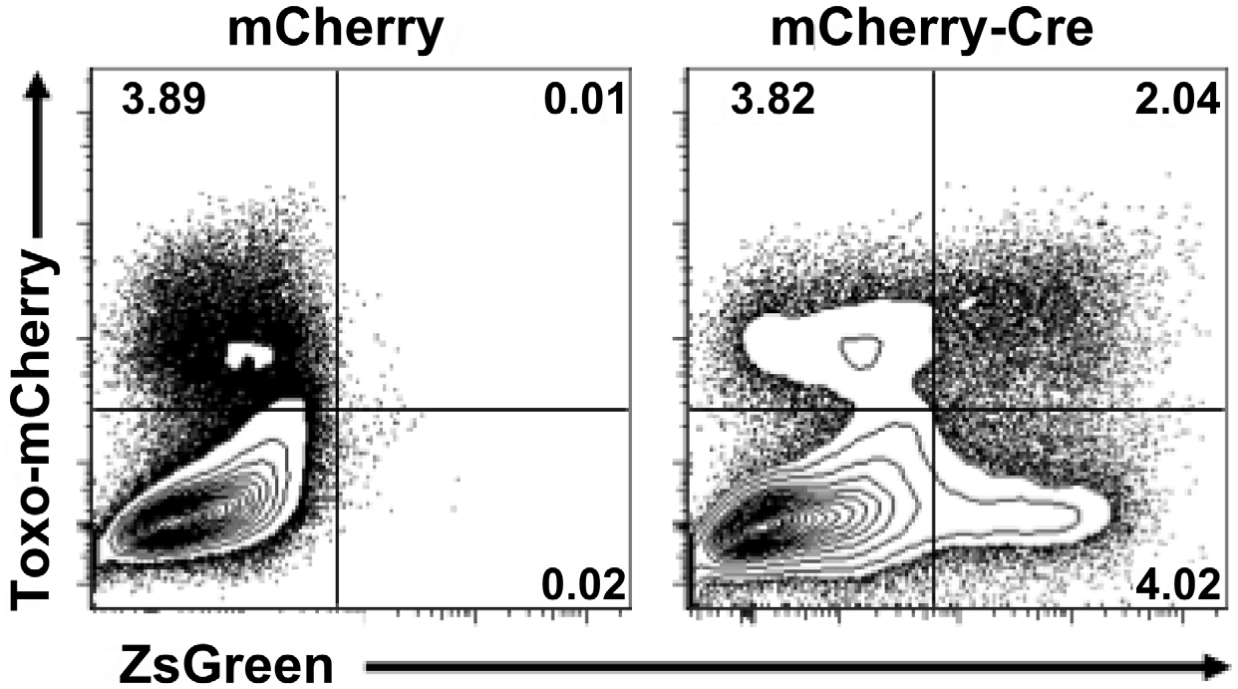
Uninfected-injected cells are 2–5 fold more common than infected-injected cells in PECs from Cre-reporter mice. As for Figure 4 except the peritoneal exudate cells (PECs) were removed at 5 dpi and were analyzed by flow cytometry. The contour plots show the level of mCherry and ZsGreen in live, Dump^−^ (CD3^−^, CD19^−^, NK1.1^−^) PECs from mice infected with Pru-mCherry (left plot) or Pru-mCherry-Cre-expressing parasites (right plot). In each plot, the upper right quadrant represents the ZsGreen^+^, mCherry^+^ cells (infected ZsGreen^+^ cells) and the lower right quadrant represents the ZsGreen^+^, mCherry^−^ cells (U-I cells). Numbers indicate the percentage of cells in that quadrant. Each contour plot represents the analysis from a single Cre-reporter mouse, and is a representative analysis of 3 animals per condition.

To better assess the ratio of ZsGreen^+^ U-I cells to ZsGreen^+^ infected cells in the Cre-reporter mice, we collected PECs at 5 dpi and analyzed them by flow cytometry for the fraction of live, Dump^−^ (CD3^−^, CD19^−^, NK1.1^−^) ZsGreen^+^ cells that were either mCherry-positive (infected) or -negative (uninfected). As expected, in mice infected with the control Pru-mCherry strain, no ZsGreen^+^ cells were observed (Figure 5, left plot). Remarkably, however, in mice infected with the Pru-mCherry-Cre strain we observed that of the ZsGreen^+^ PECs, ∼60–80% were U-I cells (Figure 5, right plot; U-I cells are ZsGreen^+^/mCherry^−^ and are in the lower right quadrant; infected ZsGreen^+^ PECS are ZsGreen^+^/mCherry^+^ and are in the upper right quadrant). Given that these inflammatory cells come from an animal which has an intact immune system, the U-I PECs could be derived from one or more of three sources: (1) cell division in invaded cells that have undergone Cre-mediated recombination, (2) aborted invasion, or (3) successful invasion events but where *Toxoplasma* is cleared from the host cell by cell-intrinsic mechanisms [19], [23]. Note that although we know from the results presented in Figure 1 that, *in vitro*, infected fibroblasts are able to divide post-invasion, we have no data to confirm or refute such a possibility in inflammatory cells *in vivo*.

**Figure 6:**
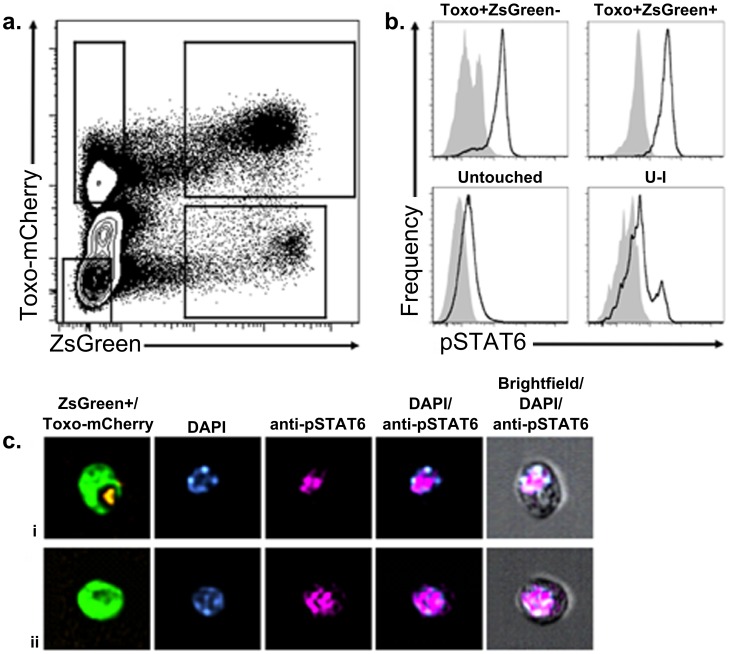
A subpopulation of uninfected-injected cells *in vivo* show phosphorylation and nuclear translocation of STAT6. Cre-reporter mice were infected with 2×10^6^ tachyzoites of RH-mCherry-Cre parasites and sacrificed at 18–20 hpi. The PECs were removed and processed in the presence of phosphatase inhibitor, stained for dead cells using Live/Dead stain, fixed with 2% PFA, and permeabilized overnight in 90% methanol. Cells were then stained for pSTAT6 and analyzed by flow cytometry. (a) Representative contour plot of mCherry and ZsGreen fluorescence of live cells to allow gating of the Untouched, Toxo^+^ZsGreen^−^, Toxo^+^ZsGreen^+^, and uninfected-injected (U-I) cell populations. (b) Representative histograms of pSTAT6 staining in each population gated in (a). (c) Representative images of pSTAT6 localization in the nucleus (DAPI) of (i) Toxo^+^ZsGreen^+^ and (ii) U-I cells as determined by high throughput fluorescence imaging on the Amnis ImageStream X. *n* = 5 mice.

To address if *in vivo* the U-I cells were manipulated in a physiologically relevant manner, we infected Cre-reporter mice with RH-mCherry-Cre parasites, and harvested the PECs 18–20 hours later. The cells were fixed, permeabilized with methanol, stained with an anti-pSTAT6 antibody, and analyzed by flow cytometry (Figure 6). As seen in Figure 6a, U-I cells are readily detected based on having a Zs-Green^+^/mCherry^−^ phenotype. Within such cells, there are two sub-populations, one in which no pSTAT6 signaling is seen, and a second, smaller population that clearly exhibits phosphorylation of STAT6 (Figure 6b). To confirm that these pSTAT6+ U-I cells also show nuclear translocation of pSTAT6, a number of cells were also examined by microscopy using the Amnis ImageStream X, which allows high throughput imaging of individual cells by brightfield and fluorescence microscopy. The pSTAT6 signal in the U-I cells was indeed found to be nuclear and indistinguishable from pSTAT6 signal in the infected ZsGreen^+^ cells (Figure 6c and Figure S1). Hence, U-I cells experience physiological changes comparable to infected cells, including in pathways with important implications for how the infection progresses.

### In the brain, U-I cells dramatically outnumber the infected cells and at least a portion are not secondary to cell division

To examine the possibility that a portion of *in vivo* U-I cells were not derived from cell division, we needed to examine cells which were: 1) commonly infected by *Toxoplasma*; 2) long-lived; 3) not prone to cell division; and 4) expressed specific markers that reflected their division history. Fortunately, encysting strains of *Toxoplasma* commonly infect neurons in the brains of mice [24] and these cells meet the remaining criteria: most neurons live as long as the animal; new neurons in adult mice only occur in very specialized regions (the dentate gyrus of the hippocampus and the subventricular zone of the lateral ventricles) where they are derived from neural stem cell precursors [25]; and for the first two weeks after neurogenesis, the nascent neuron highly expresses a protein called doublecortin (DCX) which declines in expression in the subsequent week as NeuN-staining, a marker of a mature neuron, increases [26]. Hence, a newly generated neuron expresses DCX but not NeuN, while a mature or older neuron has the opposite phenotype. Thus, to determine if we could observe U-I cells that were mature neurons (DCX^−^, NeuN^+^), we infected Cre-reporter mice intraperitoneally with the encysting Pru-mCherry-Cre strain or the control Pru-mCherry strain. At 21 dpi, we sacrificed the mice, removed the brains, and, after fixation, sectioning and appropriate staining, examined brain slices by confocal microscopy. We specifically chose to inoculate with a relatively low dose of parasites and to sacrifice the mice at 21 dpi as previous studies suggested that parasites do not arrive in the brain from i.p. inoculation until ∼4–11 dpi [27], [28]. Thus, when we examined the brain at 21 dpi, the parasite-neuron interaction at most would have occurred 17 days before our examination; this is well within the time period that a recently divided cell would still show DCX expression.

**Figure 7:**
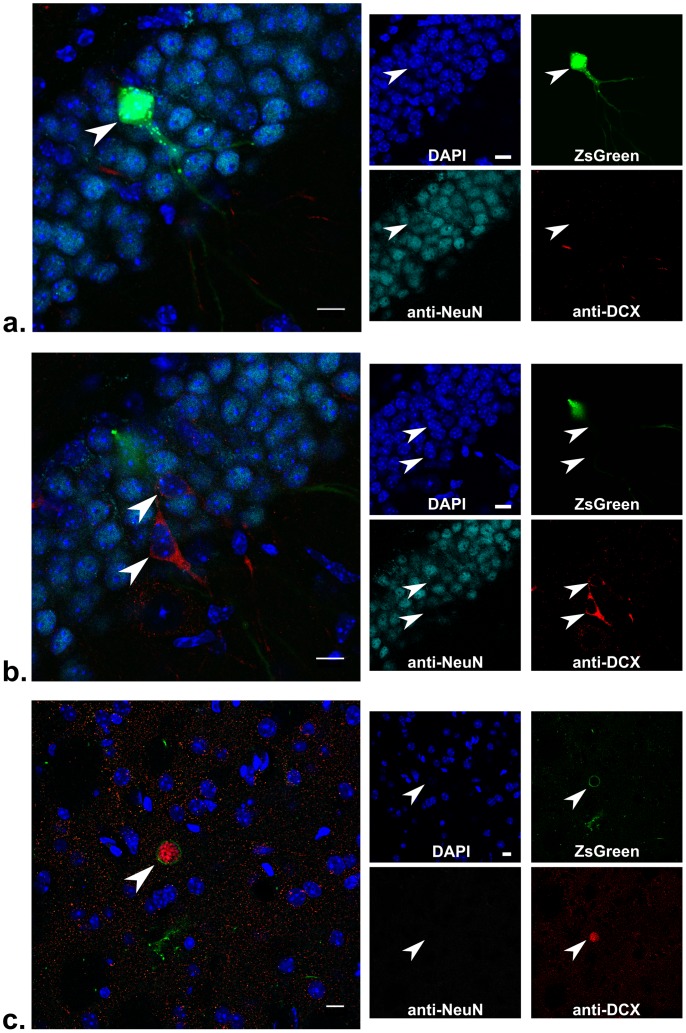
Hippocampal brain section from a Cre-reporter mouse shows an uninfected, mature neuron that is ZsGreen^+^. Cre-reporter mice were infected with 5000 tachyzoites of Pru-mcherry-Cre parasites and sacrificed at 21 dpi. The brain was removed and sectioned in 40 µm sections which were then stained for NeuN, DCX, and DAPI and examined by confocal microscopy. (a,b) Merged images from different sections in a confocal stack spanning 8 µm through the hippocampus. Individual filter images are shown to the right. Blue = DAPI, Green = ZsGreen, Red = mCherry parasites and anti-DCX antibody staining, and Cyan = anti-NeuN antibody staining. (a)Arrowhead denotes the uninfected ZsGreen^+^ cell. Scale bars = 10 µm. (b) As for (a) except arrowheads denote the two cells that stain for DCX. An expanded montage of images from the stack for (a) and (b) is shown in Supplemental Figure 1. (c) As for parts (a) and (b) except the single slice is from a different confocal stack of the same brain and shows an intracellular parasite cyst (arrowhead). Note the ZsGreen signal completely surrounding the red parasites.

Figure 7 shows a representative hippocampal brain section stained for DCX and NeuN. In Figure 7a, a ZsGreen^+^ cell that does not contain a detectable parasite stains for NeuN but not for DCX, confirming that this U-I cell has not recently undergone neurogenesis and therefore was not derived from cell division within the preceding 21 days. In Figure 7b, which is from the same hippocampal slice as that shown in Figure 7a, rare DCX-positive and NeuN-negative neurons are found, consistent with previous studies that have shown that in the adult mouse, neural stem cells in the dentate gyrus of the hippocampus continue to undergo neurogenesis at a basal rate [25]. Supplemental Figure S2 shows a montage of 16 consecutive slices from the full confocal scan of this 40 µm section in which no parasite or cyst is seen throughout the ZsGreen^+^ cell. Figure 7c shows another section stained for DCX and NeuN in which an infected ZsGreen^+^ cell that does not stain for either DCX or NeuN is seen; this serves as an example of how an infected cell appears under these staining conditions. No ZsGreen^+^ cells were seen in the brains of uninfected Cre-reporter mice or Cre-reporter mice infected with the control Pru-mCherry strain (data not shown and Supplemental Figure S3).

**Figure 8:**
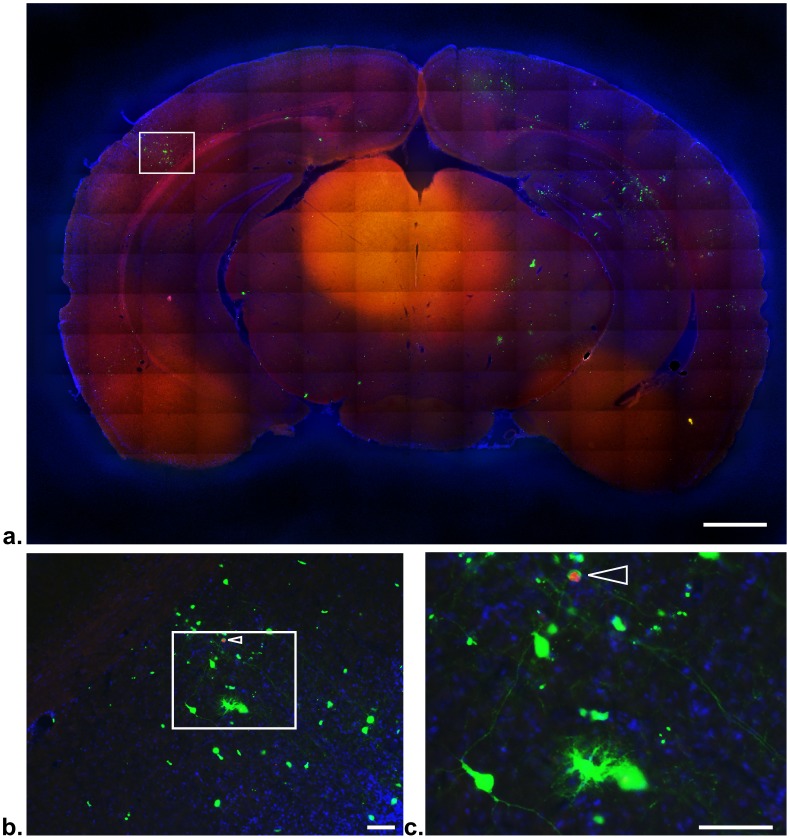
Uninfected-injected cells vastly outnumber Toxoplasma-mCherry-Cre cysts. Cre-reporter mice were infected with 5000 tachyzoites of Pru-mCherry-Cre parasites and sacrificed at 21 dpi. The brains were removed and sectioned in 40 *μ*m coronal sections which were then stained with DAPI and mounted on slides. The mounted sections were then imaged with a 10× objective on an epifluorescence microscope. Blue = DAPI, Green = ZsGreen, Red = mCherry. (a) A full coronal brain slice was reconstructed using a stitched grid of composite images. Scale bar = 1 mm (b) The boxed section of panel (a) was expanded to show the density of the ZsGreen^+^ cells. The arrowhead indicates a cyst within a host cell. Scale bar = 50 µm. (c) The boxed section of panel (b) was further enlarged to show the different morphologies and many fine processes of the ZsGreen^+^ cells. Scale bar = 50 µm.

To address the ratio of U-I cells to infected cells in the brain, we examined by fluorescence microscopy a total of 20 brain slices (8 from one infected reporter mouse and 12 from another) and counted the number of ZsGreen^+^ cells seen compared to the number of cysts seen. Strikingly, we found between 30 and 50 times the number of U-I cells compared to the number of cysts/infected cells. Figure 8 is a representative, stitched grid of composite images of a single brain slice which shows both cysts (arrowheads in Figure 8b,c) and ZsGreen^+^ cells, many but not all of which are morphologically consistent with neurons. Not surprisingly, in the mouse that had far more cysts (11 cysts/slice vs. 2 cysts/slice in the less infected animal), far more ZsGreen^+^ cells were seen, as well (∼600/slice vs. ∼60/slice, respectively). It is unlikely that we are missing significant numbers of single parasites in distant projections of neurons because we have not found any single parasites even when the sections were stained with antibodies to *Toxoplasma* specific surface antigens and examined at high magnification (data not shown.) In addition, the lack of single parasites at 21 dpi is consistent with previously published electron micrographs [29]. It also seems unlikely that ZsGreen or Cre is passing from neuron to neuron as Cre-reporter mice such as these have been extensively used to examine neuronal lineage and specific neuronal subtypes (e.g., dopaminergic neurons) with no apparent cell-to-cell transmission of the fluorescent reporter signal being noted [30], [31]. Taken together with the PECs findings, these data confirm that U-I cells can be found frequently *in vivo*, a portion are manipulated in a physiologically relevant manner, and that, in the brain of a chronically infected animal, U-I cells can arise from mechanisms other than cell division.

## Discussion

The results presented here show that *Toxoplasma gondii* tachyzoites can inject effector proteins into cells that they do not productively invade. *In vitro*, we have established that these uninfected-injected cells (U-I cells) occur with both Type I (RH) and Type II (Pru) *Toxoplasma* strains and can be identified soon after the host cells are exposed to the parasites. We have also shown *in vitro* that while some U-I cells are derived from cell division, others appear to be the result of non-productive invasion. Although the results indicated that U-I cells may be injected with less rhoptry protein than productively invaded cells, the amounts introduced are nonetheless sufficient to produce physiologically relevant changes (e.g., phosphorylation of STAT6) in these cells. *In vivo*, we have shown that U-I cells are generated in several cell types (PECs as well as in neurons), and that in the brain, at least some U-I cells have not divided within the time frame of the experiment. Given the *in vitro* data, these results are most easily explained by a similar non-productive invasion mechanism operating *in vivo*. We cannot exclude, however, the possibility that these U-I cells were infected but cleared the parasite without concomitant destruction of the infected cell. For example, autophagy has been implicated as part of the innate immune system in regards to controlling *Toxoplasma* infection [32], and is hypothesized to specifically play a role in controlling toxoplasmic encephalitis [33] though no direct evidence has shown that neurons are able to clear *Toxoplasma* via autophagy, and there are mixed reports of autophagy helping or hindering neurotropic viruses [34], [35]. Regardless of how they come to be, the results presented here show that U-I cells can exist at a surprisingly high frequency in infected animals and that a previously unknown mechanism is operating that results in rhoptry proteins being found within such cells. How long such proteins persist within these cells cannot yet be estimated and will likely vary depending on the identity of the protein. The Cre-reporter mice used here “lock in” a positive signal (i.e., once Cre has mediated the recombination, the cells will continue to express Zs-Green, even after no Cre remains) so no conclusion can be reached about how long the Cre fusion protein persists in the U-I cells. Given the right evolutionary pressure, however, proteins could evolve within *Toxoplasma* that might persist for an extended period in U-I cells and/or that lock the cell into a given state by other (e.g., epigenetic) means.

One of the most striking observations reported here is the frequency of U-I cells *in vivo*. When paired with the STAT6 data showing that the U-I cells can be activated in a similar manner to infected cells, the results beg the question of what impact these U-I cells might have on the infected host. For example, U-I cells could also represent a way of systemically manipulating the host; e.g., phosphorylation and nuclear translocation of STAT3/6 causes decreased IL-12 production from macrophages [3] and so the U-I macrophages that are pSTAT6+ could increase the number of immune cells with depressed IL-12 production. This manipulation of innate immune cells could alter the overall balance of cytokines within the host and thereby influence the delicate Th1/Th2 equilibrium of the immune response. In addition, since at least one *Toxoplasma*-specific T cell epitope is derived from a rhoptry protein [36], if the injection without invasion provides enough parasite protein for loading onto host MHC molecules, these U-I cells might be targets for the host's immune response. In turn, these cells could represent a selective advantage to the parasite, e.g., through providing antigenic decoys (the immune response becomes directed at the U-I cells and not the productively invaded cells) or they might favor the host through providing more antigenic priming to cells of the adaptive immune response. In either case, the targeting of U-I cells could contribute substantially to the pathogenesis of the disease

The abundance of U-I cells in the brain was particularly remarkable. In part, this is likely because neurons which either cleared the parasite or were never invaded will potentially survive for the rest of the mouse's life, unlike many peripheral immune cells (e.g. macrophages) which have a finite life span. This accumulation of affected cells might help explain the notable and well-documented changes in behavior in rodents chronically infected with *Toxoplasma* [37], [38], a phenomenon that previously has been difficult to explain based on the very small numbers of parasites (cysts) actually present in the chronically infected brain [38].

The ability to inject cells that are not productively invaded may not be limited to *Toxoplasma*. Given the similarity in the invasion process between many members of the phylum Apicomplexa, it would not be surprising to find that other species also utilize this mechanism for manipulating the host environment. For the malaria parasite, *Plasmodium spp.*, for example, the ability to manipulate cells in which they cannot productively invade or grow (e.g. leukocytes) might represent a potent means of directly influencing the immune response. Ultimately, the finding that uninfected cells can be modulated by *Toxoplasma* will have a significant impact on how the global effects of infection with this parasite are analyzed, and if this ability occurs in other intracellular pathogens, it may lead to a shift in the overall paradigm of the host-pathogen interaction.

## Materials and Methods

### Ethics statement

This study was carried out in strict accordance with the Public Health Service Policy on Humane Care and Use of Laboratory Animals and AAALAC accreditation guidelines. The protocol was approved by Stanford University's Administrative Panel on Laboratory Animal Care (Animal Welfare Assurance # A3213-01, protocol # 9478) UPenn: Multiple project assurance # A3079-01. All efforts were made to minimize suffering.

### Generation and selection of Cre- and -β-lacatmase-secreting *Toxoplasma*

The parental strain used for encysting *Toxoplasma* was the type II Pru*Δhpt* in which the endogenous gene for hypoxanthine xanthine guanine phosphoribosyl transferase (HPT) has been deleted. All strains were propagated in human foreskin fibroblasts (HFFs). The vector expressing the selectable *HPT* marker and the epitope-tagged rhoptry protein fused to Cre recombinase (*pToxofilin-Cre*) has been previously described [6]. In addition to the *pToxofilin-Cre* plasmid, a second plasmid containing the coding sequence for mCherry, flanked by the *GRA2* promoter and 5′-UTR and the *GRA2* 3′-UTR, was co-transfected into the parasites. The parental strain for all non-encysting parasites was the type I strain RH*Δhpt* [39]. The generation of RH-Cre and the vector expressing *HPT* and a toxofilin:β-lactamase fusion have been previously described [20]. In addition to the *pToxofilin-β-lactamase* plasmid, a second plasmid containing the coding sequence for mCherry flanked by the *GRA1* promoter and 5′-UTR and *GRA2* 3′-UTR, was co-transfected into the parasites. To generate mCherry^+^ parasites expressing the respective toxofilin fusion protein, the parental parasites were electroporated with the appropriate plasmids, linearized upstream of the relevant expression cassettes prior to electroporation. As previously described, the parasites were then subjected to several rounds of selection for expression of HPT using medium containing 25 µg/ml mycophenolic acid and 50 µg/ml xanthine before being cloned by limiting dilution [40]. Single cell clones that were HPT^+^ and mCherry^+^ and confirmed to express the appropriate toxofilin fusion protein were then tested for efficacy in causing Cre-mediated recombination in a Cre-reporter cell line and Cre-reporter mice [6], or for the ability to cleave the substrate CCF2-AM [20] to verify secretion of a functional toxofilin:Cre or toxofilin:β-lactamase fusion protein, respectively. For the encysting Pru-Cre strain, a control parasite strain was also selected that both expressed mCherry and had the selectable marker but, unlike the Pru-Cre strain, expressed a truncated form of the Cre fusion protein, as determined by western blot analyses (data not shown). This truncated fusion protein was inactive as infection of either the Cre-reporter cells or mice with this control strain did not result in Cre-mediated recombination (data not shown).

### Evaluation of rhoptry secretion using Cre-reporter cells

For detection of Cre, specially engineered 10T ½ fibroblasts were used [6]. These have a cassette for eGFP downstream of a translational stop signal flanked by *loxP* sites so that upon Cre-mediated recombination, the eGFP is expressed. These Cre-reporter cells were plated on glass coverslips and infected 24 h later with syringed-released *Toxoplasma*-Cre parasites at an MOI of 0.5, washed with 1× phosphate-buffered saline (PBS) 2 hours after incubation, and returned to the 37°C incubator until the following day when the infected monolayers were fixed with 3.5% formaldehyde. For the *Toxoplasma*-Cre strain that did not express mCherry, after fixation the cells were permeabilized and blocked for 2 hours in 1× PBS supplemented with 0.1% (v/v) Triton-X 100 (TTX; Sigma) and 3% (w/v) bovine serum albumin (BSA). Cells were then stained with the mouse anti-SAG1 monoclonal antibody DG52 [41] (dilution 1∶8,000) followed by the AlexaFluor647-goat-anti-mouse antibody (Molecular Probes, 1∶2000). After fixation and staining, if appropriate, coverslips were mounted on slides using Vectashield Mounting Media for Fluorescence with DAPI (Vector laboratories). For Figure 1a and b, slides were viewed with a Leica TCS SPE confocal microscope. All digital images were obtained using Leica Application Suite, Advanced Fluorescence. For Figure 1c, slides were viewed on an Olympus BX60 upright fluorescence microscope, and images were obtained using Image-Pro Plus. All images shown in a given figure, using a given microscope and camera, and with a given color were obtained and processed using identical parameters.

### Evaluation of rhoptry secretion in human foreskin fibroblasts (HFFs) using the β-lactamase assay

HFFs were plated on 24-well glass bottom plates (MatTek Corporation, #P24G-1.5-13-F) the day prior to incubation with *Toxoplasma*-β-lactamase parasites. The β-lactamase assay was carried out essentially as described previously [20]. In brief, prior to live imaging, *Toxoplasma*-β-lactamase parasites were syringed-released and incubated with HFFs at an MOI of 0.5 for 2 hours in a 37°C incubator. After incubation, the cells were washed once with PBS, then 300 µl of CDMEM was added after which 60 µl of 6× CCF2-AM (Invitrogen K1085) was added to the wells and allowed to equilibrate as directed by the manufacturer's protocol. The live cells were then imaged with a LSM Meta Confocal Microscope (Neuroscience Microscopy Service, Stanford University, Stanford, CA). To determine if the CCF2 had been cleaved, the cultures were excited with a blue diode 405 nm laser and the detectors set for 410–450 nm for coumarin (cleaved) and 493–550 nm for fluorescein (uncleaved). To visualize parasites, the cultures were excited with a 561 nm laser with detectors set to pick up emissions greater than 600 nm. All images were obtained using Zen 2009. For flow cytometry assay, cultures were prepared as detailed above except that tissue-culture-treated T25s were used. After 30 minutes of substrate loading, cells were washed with cold PBS, and then trypsinized at 25°C. Cells were then kept on ice until they were analyzed on a Becton-Dickinson LSR II that has been modified to include a UV laser and has both a 405 nm and 561 nm laser. MCherry expression was used to determine if cells were infected and detection in the cascade blue channel was used to determine whether or not the CCF2 had been cleaved. Analysis was done using FlowJo v. 9.4.9 software.

### Evaluation of the effect of *Toxoplasma* infection on uninfected HFFs

Confluent HFF cultures plated on coverslips were infected with 10^4^ non-encysting parasites (RH*Δhpt*) (MOI ∼0.02 ). At two hours post infection (hpi), the cells were washed twice with 1× PBS and then left for 16 hours in CDMEM. At 18 hpi, infected monolayers were washed once with cold 1× PBS and then fixed with cold methanol for 10 minutes at −20°C. After washing the cells with 1× PBS supplemented with 0.2% (v/v) TTX, they were blocked for 2 hours with 1× PBS supplemented with 3% (v/v) goat serum and 0.2% (v/v) TTX. After washing cells with 1× PBS supplemented with 0.2% TTX, they were incubated overnight at 4°C with primary antibodies (rabbit anti-pSTAT6 (Cell Signaling Technologies #9361S) at 1∶100 and mouse anti-SAG1 monoclonal antibody DG52 [41] at 1∶30,000) in 1× PBS supplemented with 3% (v/v) goat sera and 0.2% TTX. The cells were then washed with 1× PBS supplemented with 0.2% TTX and incubated with species-appropriate Alexa Fluor-conjugated secondary antibodies (Molecular Probes, 1∶2000) at room temperature for 1 hour. Stained coverslips were then mounted onto slides as described above. Images were taken using QCapture v.3.1 software and using an Olympus BX60 upright fluorescent microscope.

### Infection of Cre-reporter mice

Cre-reporter mice (background C57B6) were purchased from Jackson Laboratories (stock # 007906) and bred in a Specific Pathogen Free, AAALAC, Int.-approved, conventional facility. In the *Rosa26* locus of these mice is a cassette for ZsGreen downstream of a transcriptional stop that is flanked by *loxP* sites so that only after Cre-mediated recombination will the mouse's cells express ZsGreen [22]. One to 5 days prior to infection the mice were transferred to the biohazard suites. For inoculation, *Toxoplasma* strains were grown in HFFs, and intracellular parasites were syringe-released, and counted on the day of infection. The parasites were diluted to appropriate inoculum sizes in sterile, serum-free 1× PBS. Mice were injected intraperitoneally (i.p.) with a total volume of 200 µl containing the appropriate number and type of parasites. For the Cre recombinase injection, 2 days post infection (dpi) with parasites, the infected mouse was injected with 20 units of Cre recombinase (NEB, M0298L) in a 200 µl solution of 1× serum-free PBS and Cre reaction buffer.

### Collection of peritoneal exudate cells (PECs)

At 4 or 5 dpi, infected mice were euthanized by CO_2_, and PECs were collected by peritoneal lavage with 5–8 ml of cold 1× PBS. For the PECs that were to be examined by microscopy, all samples were treated with 1 ml of ACK lysis buffer (Invitrogen) for 3 minutes and then resuspended in 2 ml of CDMEM. About 600 µl of this suspension were then placed onto poly-L-lysine coated glass coverslips and placed in a 37°C incubator. The cells were allowed to settle for 30 minutes, after which the coverslips were washed with 1× PBS, then fixed with 2.5% formaldehyde for 15–20 minutes. After being mounted on slides with Vectashield Mounting Media for Fluorescence with DAPI (Vector laboratories), slides were viewed with a 100× oil immersion lens on an Olympus BX60 upright fluorescence microscope. All digital images were obtained by using Image-Pro Plus and all images shown in a given figure and with a given color were obtained and processed using identical parameters. The PECs used for flow cytometry analysis came from mice different from those used for microscopy. These PECs were first incubated for 10 min in FcBlock containing Normal Rat Serum IgG and Live/Dead (Invitrogen) as a viability marker. Cells were then stained for surface markers for 20 minutes on ice. The following antibodies were used for staining: Pacific Blue-anti-mouse CD3 clone 17A2 (Biolegend), eFluor 450-anti-mouse CD19 clone 1D3 (eBioscience), and Pacific Blue-anti-mouse NK1.1 clone PK136 (Biolegend). The PECs were kept on ice until analysis on an LSR Fortessa. The data were collected with FACSDiva software and analyzed with FlowJo software.

### Evaluation of pSTAT6 signal in PECs

Cre-reporter mice were infected i.p. with 2×10^6^ parasites. At 20 hours post infection PECs were harvested using ice-cold Phospho-wash (PBS containing Phosphatase Inhibitor Cocktail 2 (Sigma, P5726) and Roche complete protease inhibitor (Roche)). Cells were immediately centrifuged, resuspended in 500 µL Phospho-wash containing Live/Dead aqua stain (Invitrogen, L34957), and incubated for 10 minutes on ice. Cells were rinsed with 500 µl Phospho-wash, and then fixed in 2% PFA containing Phosphatase Inhibitor Cocktail 2 for 20 minutes on ice. Cells were again rinsed with 500 µl Phospho-wash, resuspended in 400 µl 90% methanol in PBS, and incubated overnight at −20°C. After incubation, cells were rinsed with FACS buffer and blocked with FcBlock (BD Pharmingen) and normal rat serum for 10 minutes. Cells were then stained on ice for 4 hours using AlexaFluor647-anti-pSTAT6 pY641 (clone: J71-773.58.11, BD Pharmingen) or a Rat IgG1k Alexa Fluor 647 isotype control (BD Pharmingen). Cells were then washed and analyzed by flow cytometry. For analysis of pSTAT6 localization using the Amnis ImageStream X, cells were stained with DAPI for 10 minutes.

### Evaluation of infected brain cells

At 21–29 dpi, mice were anesthetized with a ketamine/xylazine cocktail (24 mg/ml and 48 mg/ml, respectively) and intracardially perfused with heparin (10 U/ml) in a 0.9% saline solution followed by fresh 4% paraformaldehyde (Sigma P6148). Brains were then collected and drop-fixed in 4% paraformaldehyde for 24 hours, cryoprotected in 30% sucrose in 1× PBS, and stored in 30% sucrose at 4°C until sectioned. Free-floating coronal or sagittal sections (40 µm) were cut on a sliding freezing microtome (Microm HM 430) and stored at 4°C in cryoprotective medium (0.05 M sodium phosphate buffer containing 30% glycerol and 30% ethylene glycol). Sections were selected at random and immunostained with primary antibodies (anti-NeuN clone A60, biotin conjugated (Millipore, 1∶200) and goat polyclonal anti-Doublecortin C-18 (Santa Cruz, 1∶200)) or not stained as appropriate. Species-appropriate or streptavidin Alexa Fluor-conjugated secondary antibodies were used (Molecular Probes, 1∶200). Brain sections were mounted on slides using Vectashield Hardmount with or without DAPI (Vector laboratories) and viewed with a 40× oil lens on a Leica TCS SPE confocal microscope, a 40× oil lens on a Zeiss LSM 510 meta scanning confocal, or a 10× lens on an upright widefield fluorescence microscope (Zeiss AxioImager M1 with CCD camera). Confocal images were obtained using Leica Application Suite, Advanced Fluorescence or Zen 2009 (Zeiss) and upright fluorescent images were obtained using AxioVision software including Multichannel, MosaiX, Autofocus, and Mark and Find modules. All images shown in a given figure and with a given color were obtained using identical parameters. Images were analyzed and processed using ImageJ/FIJI.

## Supporting Information

Figure S1 **Representative images of pSTAT6 localization in infected or uninfected ZsGreen^+^, pSTAT6^+^ cells.** The first seven images of live cells taken by the Amnis ImageStreamX of ZsGreen^+^,pSTAT6^+^ cells which were (a) infected (mCherry^+^) or (b) uninfected (mCherry^−^) are shown. The cells are from a single mouse and they were collected 20 hpi with RH-mCherry-Cre tachyzoites. 40× magnification.
(TIF)

Figure S2 **No parasites or cysts are detectable throughout the hippocampal section containing an uninfected ZsGreen^+^ cell.** Cre-reporter mice were infected with 5000 tachyzoites of Pru-mCherry-Cre parasites. This image is a montage of 16 serial slices generated in viewing the hippocampal section seen in Figure 6 (a) and (b). Blue = DAPI, Green = ZsGreen, Red = mCherry parasites and anti-DCX antibody staining, and Cyan = anti-NeuN antibody staining. Scale bar = 10 µm.
(TIF)

Figure S3 **No ZsGreen^+^ cells are found in brain sections of mice infected with control Pru-mCherry strain.** Cre-reporter mice were infected with 5000 tachyzoites of Pru-mCherry or Pru-mCherry-Cre and sacrificed at 4 weeks post infection. The brains were removed and sectioned in 40 µm sections which were mounted with DAPI and examined by confocal microscopy. Blue = DAPI, Green = ZsGreen, Red = mCherry. (a) Representative montage of composite images from a section from a Pru-mCherry infected mouse. The large red center represents the mCherry signal from a tissue cyst containing many bradyzoites. An additional 3 slices are shown compared to (b) to verify that ZsGreen signal was not missed in sections that that did not contain parasites. (b) Representative montage of composite images from a section from a Pru-mCherry-Cre infected mouse. Sections were mounted and imaged on the same day, using the same microscope and settings for each channel. Scale bar = 10 µm.
(TIF)
