# Supplementary figures and images for: Toxoplasma Co-opts Host Cells It Does Not Invade
Source: PLoS Pathog. 2012 Jul 26;8(7):e1002825. doi: 10.1371/journal.ppat.1002825 (PMC3406079; doi:10.1371/journal.ppat.1002825)

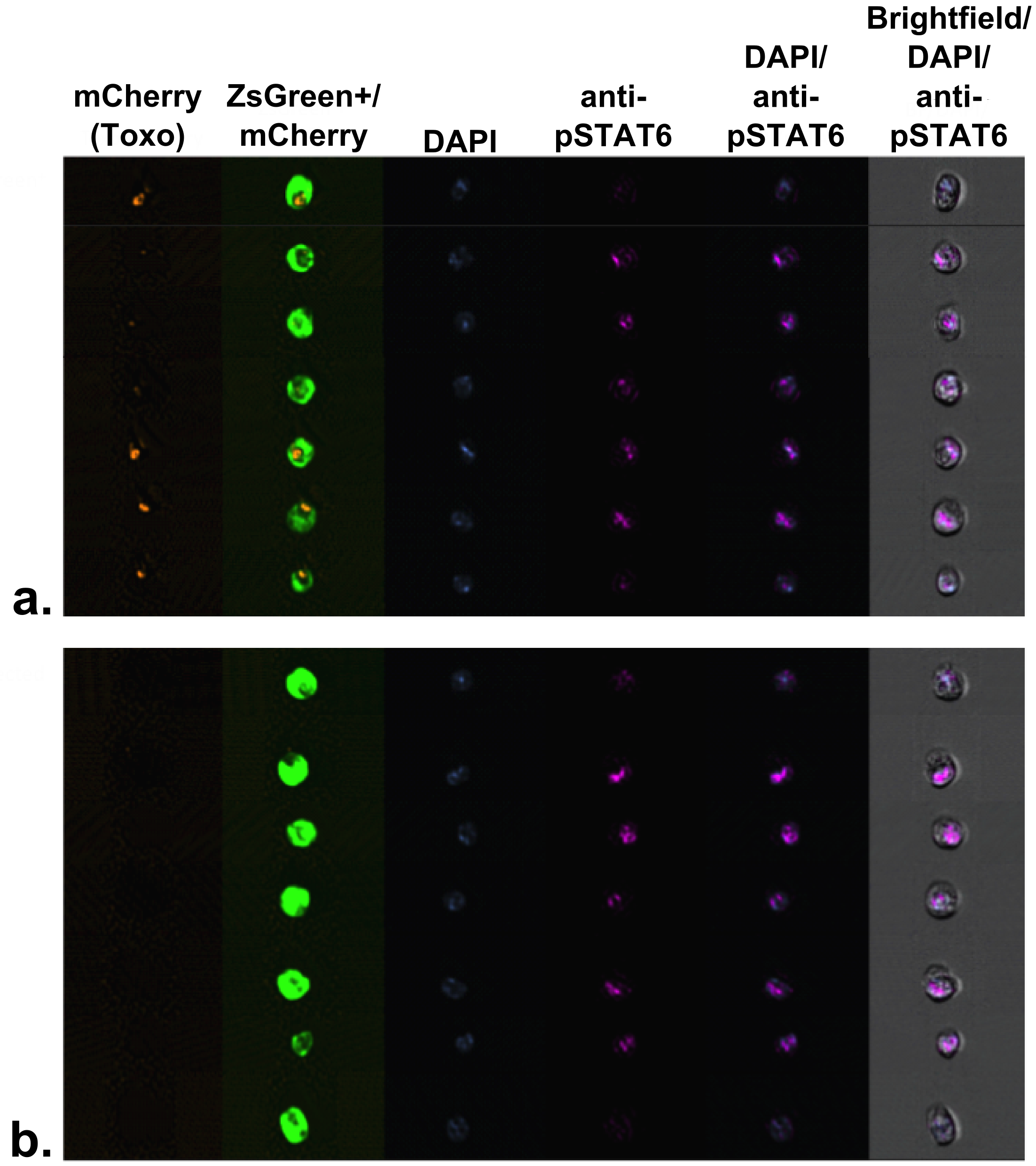

Supplement: Figure S1 — Representative images of pSTAT6 localization in infected or uninfected ZsGreen+, pSTAT6+ cells. The first seven images of live cells taken by the Amnis ImageStreamX of ZsGreen+,pSTAT6+ cells which were (a) infected (mCherry+) or (b) uninfected (mCherry−) are shown. The cells are from a single mouse and they were collected 20 hpi with RH-mCherry-Cre tachyzoites. 40× magnification. (TIF) [file ppat.1002825.s001.tif]

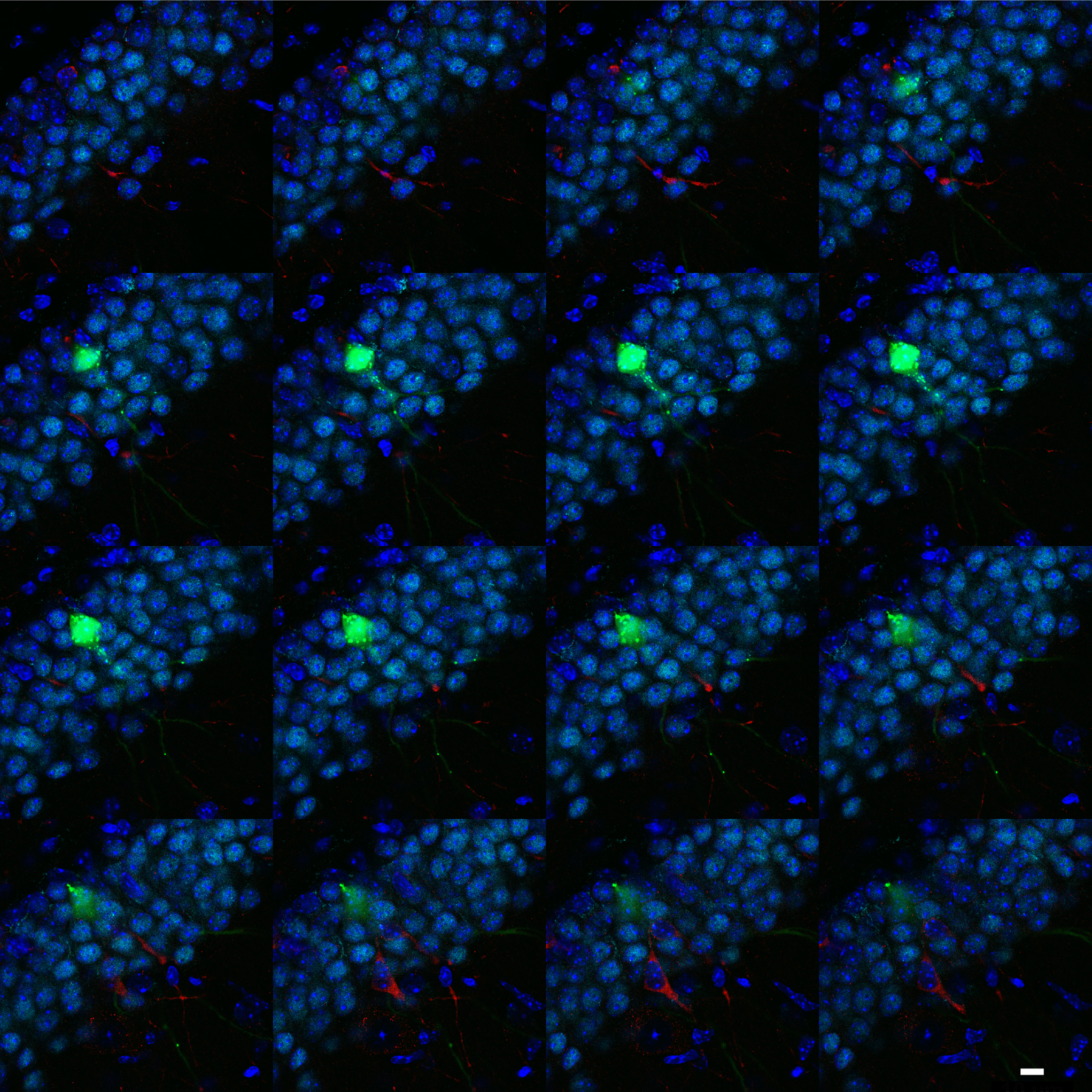

Supplement: Figure S2 — No parasites or cysts are detectable throughout the hippocampal section containing an uninfected ZsGreen+ cell. Cre-reporter mice were infected with 5000 tachyzoites of Pru-mCherry-Cre parasites. This image is a montage of 16 serial slices generated in viewing the hippocampal section seen in Figure 6 (a) and (b). Blue = DAPI, Green = ZsGreen, Red = mCherry parasites and anti-DCX antibody staining, and Cyan = anti-NeuN antibody staining. Scale bar = 10 µm. (TIF) [file ppat.1002825.s002.tif]

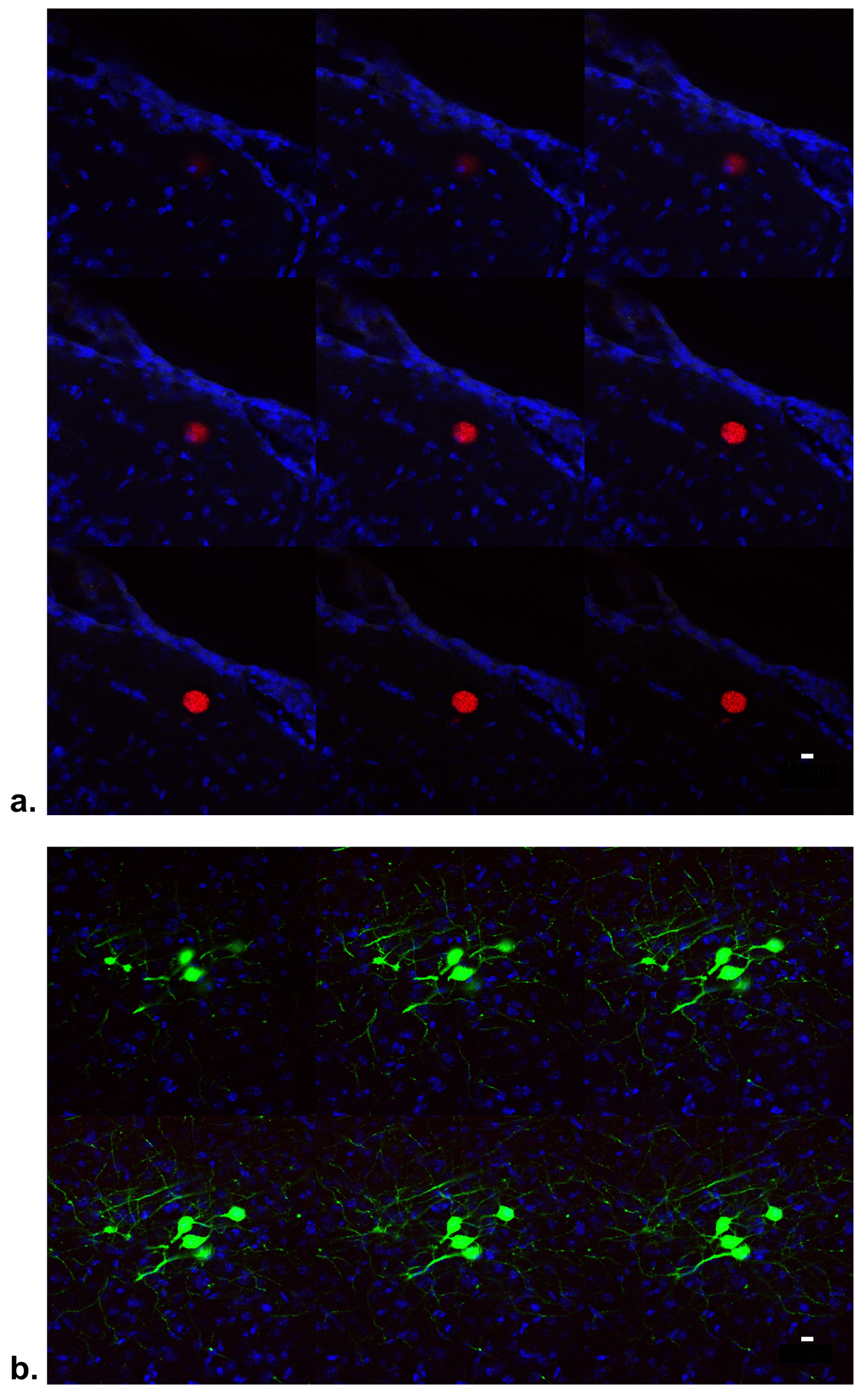

Supplement: Figure S3 — No ZsGreen+ cells are found in brain sections of mice infected with control Pru-mCherry strain. Cre-reporter mice were infected with 5000 tachyzoites of Pru-mCherry or Pru-mCherry-Cre and sacrificed at 4 weeks post infection. The brains were removed and sectioned in 40 µm sections which were mounted with DAPI and examined by confocal microscopy. Blue = DAPI, Green = ZsGreen, Red = mCherry. (a) Representative montage of composite images from a section from a Pru-mCherry infected mouse. The large red center represents the mCherry signal from a tissue cyst containing many bradyzoites. An additional 3 slices are shown compared to (b) to verify that ZsGreen signal was not missed in sections that that did not contain parasites. (b) Representative montage of composite images from a section from a Pru-mCherry-Cre infected mouse. Sections were mounted and imaged on the same day, using the same microscope and settings for each channel. Scale bar = 10 µm. (TIF) [file ppat.1002825.s003.tif]
